# Influence of the Presence of Disulphide Bonds in Aromatic or Aliphatic Dicarboxylic Acid Hardeners Used to Produce Reprocessable Epoxidized Thermosets

**DOI:** 10.3390/polym13040534

**Published:** 2021-02-11

**Authors:** Chiara Di Mauro, Alice Mija

**Affiliations:** Institut de Chimie de Nice, Université Côte d’Azur, UMR CNRS 7272, 28 Avenue Valrose, 06108 Nice, France; Chiara.DI-MAURO@univ-cotedazur.fr

**Keywords:** dicarboxylic acids, epoxidized vegetable oils, dynamic disulphide diacid hardeners, reactivity, reprocessing, thermomechanical properties

## Abstract

The design of polymers from renewable resources with recycling potential comes from economic and environmental problems. This work focused on the impact of disulphide bonds in the dicarboxylic acids reactions with three epoxidized vegetable oils (EVOs). For the first time, the comparison between aromatic vs. aliphatic dicarboxylic acids, containing or not S–S bonds with EVOs was discussed and evaluated by dynamic scanning calorimetry. The obtained thermosets showed reprocessability, by the dual dynamic exchange mechanism. The virgin and reprocessed materials were characterized and the thermomechanical properties were compared. The thermosets derived from EVOs with high epoxy content combined with aromatic diacids containing disulphide bridges showed high glass transition values (~111 °C), high crosslink densities and good solvent stability.

## 1. Introduction

The development of vitrimers, incorporating exchangeable chemical bonds, has grown in recent years, thanks to their characteristics of having a hybrid behaviour, acting like thermoplastics above critical temperatures, and as thermosets below this transition temperature. The excellent mechanical properties of these materials combined with the ability to be recyclable and reprocessable are increasing the attention on this relatively new class of materials as alternatives to conventional resins. In 2011, Leibler et al. [1] showed these capabilities for the first time in epoxy-based materials, using diglycidyl ether of bisphenol a (DGEBA) as an epoxy matrix. In the following years, other research groups developed vitrimers and vitrimer-like materials by expanding the different mechanisms of possible reactions: from carboxylate transesterification reactions [2,3] to the metathesis of disulphide bonds [4,5,6,7,8,9] or to olefin metathesis [10,11].

The demand for recyclable materials is permanently increasing, together with the growing interest to develop new materials from renewable resources, with an economical potential and at least analogous performances to those obtained from fossil raw materials. Several bio-based epoxidized monomers have been used to obtain vitrimers: isosorbide, [12] eugenol [13,14] or epoxidized natural rubber (ENR). [7,15]. Recently, Memon et al. [16] reported the synthesis of disulphide-containing epoxy resins, the bis (4-glycidyloxyphenyl)disulphide, crosslinked with methyl hexahydrophthalic anhydride (MHHPA), producing resins having excellent weldability and reprocessability, low relaxation times, high *T*_g_ and thermal stability. Among other promising renewable resources, vegetable oils are considered an important class of renewable resources thanks to their high availability, low cost and versatility. The double bonds of these oils can be easily converted into new polymerizable functions. One of the most advantageous is the epoxidation in which the double bonds are converted into oxirane rings. Altuna et al. [17] reported that the epoxidized soybean oil (ESO) crosslinked with an aqueous citric acid solution can be used as a building block to generate self-healing polymer networks. For a long time, epoxidized linseed oil (ELO) has been used as the matrix in curing reactions with acids. Supanchaiyamat et al. [18] obtained thermosets using Pripol 1009, while our team [19] synthesized a diacid hardener starting from maleic anhydride and dipropylene glycol. 

In this study, for the first time to our knowledge, the influence of the hardener structure, i.e., aromatic vs. aliphatic dicarboxylic acids with or without S–S linkages was investigated in order to produce reprocessable bio-based thermosets, using epoxidized vegetable oils (EVOs) as epoxy monomers. Four types of diacid hardeners were selected, with analogous chemical structures, two aromatic and two aliphatic, and their impact on curing kinetics was firstly discussed. Moreover, the properties of virgin and reprocessed materials were evaluated, compared and discussed in function of the crosslinker structure regarding the presence of S–S linkages. Three epoxidized vegetable oils were studied: epoxidized perilla oil (EPLO), epoxidized safflower oil (ESFO) and epoxidized linseed oil (ELO) (as reference). The reactivity and the kinetic of the EVOs/hardeners curing formulations were analysed by DSC and the thermo-mechanical properties of virgin and reprocessed thermosets were investigated by DSC, TGA and dynamic mechanical analyses (DMAs). 

## 2. Materials and Methods

### 2.1. Materials

**Scheme 1:**
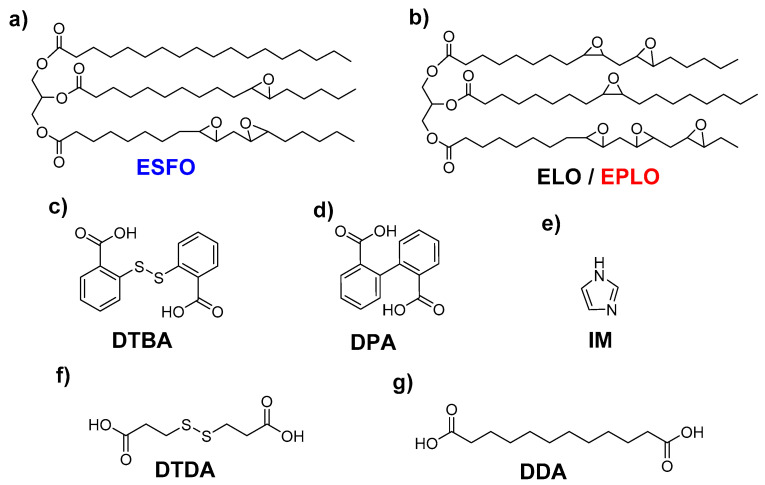
Chemical structures of the selected epoxy monomers (**a**,**b**), hardeners (**c**,**d**,**f**,**g**) and initiator (**e**).

The bio-based epoxidized monomers included: epoxidized linseed oil (ELO) (b) obtained from Valtris Chemicals (Greater Manchester, England); epoxidized safflower oil (ESFO) (a) and epoxidized perilla oil (EPLO) (b) provided by SPECIFIC POLYMERS (Castries, France). Their chemical structures and characteristics are reported in Table S1 and Scheme 1.

The hardeners and the initiator were purchased from Sigma-Aldrich (St. Louis, MO, USA) and used as received without further purification: 2-[(2-carboxyphenyl) disulfanyl] benzoic acid (2,2′-dithiodibenzoic acid, DTBA) (c), 2-(2-carboxyphenyl)benzoic acid (diphenic acid, DPA) (d), 3-(2-carboxyethyldisulfanyl)propanoic acid (3,3′-dithiodipropionic acid, DTDA) (f), dodecanedioic acid (DDA) (g) and imidazole (IM) (e).

### 2.2. Sample Preparation

The polymer networks were prepared by reacting the EVOs with diacid hardeners in different R_e/a_ functional ratios epoxy groups/acid groups: R_e/a_ = 2 (e/a = 1/0.5), R_e/a_ = 1.25 (e/a = 1/0.8) and R_e/a_ = 1 (e/a = 1/1). In all the formulations, IM was used as the initiator as we recently showed its good selectivity on epoxy/acid copolymerization and also the positive effect on the thermoset’s recyclability [20,21,22,23,24]. According to previous results [21], the initiator was used in 1 wt.% of the total amount of the reactive mixture, as the optimal amount for curing. In the EVOs reactions with diacid hardeners, the epoxide was considered as monofunctional according to Dusek et al. [25]. The mixture preparation was done by heating the selected EVOs at 80 °C to decrease its viscosity. Then, the initiator and hardener were introduced and mixed until homogenization. 

The curing process conditions, given in Table S2, were established by preliminary DSC analyses. For the reprocessing procedure, the crosslinked thermosets were reduced to a small piece and thereafter pressed in a hot Carver manual press. The temperature and pressure (Table S2) were selected in order to obtain the optimal recyclability and the results are displayed in Table S3.

### 2.3. Methods

*Differential scanning calorimetry (DSC)* experiments were carried out on a Mettler-Toledo DSC 3 apparatus controlled by STAR^©^ Software developed by Mettler-Toledo. The copolymerization reactions were directly performed in the DSC pans, by simple heating. Samples of 7–10 mg were placed into 40 µL aluminium crucibles. The different mixtures were reacted under non-isothermal conditions at the heating rate *β* of 2.5, 5, 10 and 20 °C·min^−1^ in the temperature range of 25–230 °C. The glass transition (*T_g_)* of the thermosets was determined by 2 cycles of a heating–cooling program at 10 °C·min^−1^.

*Thermogravimetric analyses (TGA)* were carried out on a Mettler-Toledo TGA 2. Samples of about 10 mg were placed into 70 µL alumina pans. The bio-based resins were heated at 10 °C·min^−1^ from 25 to 1000 °C under 150 mL.min^−1^ air flow.

*Dynamic mechanical analyses (DMAs)* were performed using a Mettler-Toledo DMA 1 instrument, equipped with the STAR^©^ software. The analysed samples with rectangular dimensions of 30 × 7 × 2 mm^3^ (length × width × thickness) were analysed 3 times and the obtained results averaged. The analyses were operated in tension method, and the elastic modulus values (*E′*) and damping factors (tan *δ*) were collected at 3 °C·min^−1^ heating rate from −50 to 170 °C and 1.0 Hz frequency. 

Crosslinking density was calculated by the help of Equation (1):

(1) $$\nu =\;\frac{E^{\prime }}{3RT}\;\;$$

where *E’* is the storage modulus of the thermoset in the rubbery plateau region at *T_g_* +50 °C, *R* is the gas constant and *T* is the absolute temperature in Kelvin. 

*Swelling experiments* were performed by immersing in trichlorobenzene the thermosets samples of 5 × 7 × 1.5 mm^3^ (around 1 g of sample in 30 mL of solvent). To test the solvent stability in function of temperature, the samples were immersed in closed vials and heated from 100 to 180 °C by 20 °C steps [8]. Each of these temperatures, were kept constant for 2 h.

The swelling experiment at room temperature was performed for 32 h. The swelling ratio *Q* was calculated according to Equation (2):

(2) $$Q=\frac{m_{s}-\;m_{u}}{m_{u}}$$

where *m_s_* and *m_u_* are the swollen and unswollen mass of the sample. The same procedure was performed for virgin and reprocessed materials. 

A piece of each material (virgin and reprocessed resins, 5 × 7 × 1.5 mm^3^ and around 1 g) were immersed in toluene (30 mL of solvent) for two days and the weights followed over time.

*Density calculation:* the densities of the samples were determined by measuring the volume of each sample and its weight was obtained using a Mettler Toledo ML 3002T precision balance (±0.0001 g).

## 3. Results and Discussion

### 3.1. Reactivity Studies in Function of the Crosslinker Structure

The influence of the dicarboxylic acid hardeners’ structure on the copolymerization reaction with the three EVOs was analysed by dynamic DSC studies. For DDA, due to its poor immiscibility with the EVOs, it was not possible to properly evaluate its reactivity by DSC. Therefore, the EVO formulations with DDA were discarded for DSC curing study. 

In the first stage of the study, the functional ratio R_e/a_ was investigated, in order to find its optimal value for each formulation. As described in the samples preparation section, the three EVOs were combined with DTBA, DPA or DTDA at the ratios of 1, 1.25 and 2 and their reactivity was analysed by dynamic DSC. The results are given in Figures S1–S3 and Tables S4–S6. From these results, we notice that the higher reaction enthalpies were obtained for R_e/a_ = 1, with the exception of the ESFO/DTBA system, thus this ratio was selected as optimal and used for the future studies. This result supports the reported investigations where the equimolar ratio R_e/a_ = 1 gave the optimal results in terms of reactivity [22,24].

**Figure 1:**
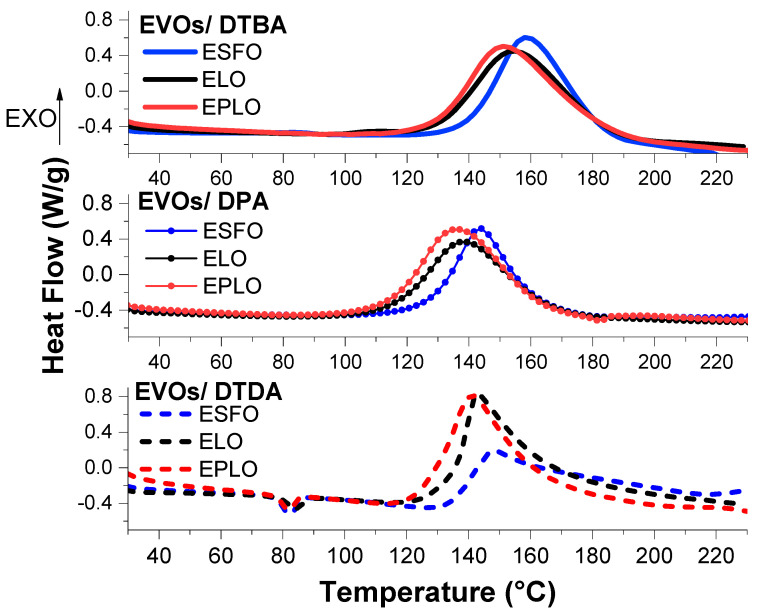
DSC thermograms during the process of curing epoxidized vegetable oils (EVOs) with DTBA and DPA aromatic hardeners or with the DTDA aliphatic crosslinker in R_e/a_ = 1. Heating at 10 °C/min from 25 to 250 °C. Each EVO’s curve is represented in a specific colour: red for EPLO, black for ELO and blue for ESFO

Figure 1 shows the comparison of the three EVOs reacted with the crosslinkers, at R_e/a_ = 1. We can firstly notice that the EVOs with higher epoxy content exhibit the onset of reaction shifted to lower temperatures. In all the analysed formulations, EPLO, with 6.50 meq·g^−1^ epoxy content, displayed the higher reactivity and low temperatures of crosslinking compared to ELO systems. The ELO (5.61 meq·g^−1^) reference is of intermediate reactivity between ESFO (5.06 meq·g^−1^) and EPLO. 

An interesting result comes from the comparison between the two aromatic crosslinkers, DTBA and DPA, is that these have a very similar structure, with the carboxylic positions in *o* and *o’* at the aromatic ring. As we can see in Figure 1 and Table 1, the aromatic crosslinker containing the S–S bond induces the higher reactivity, in the three EVOs formulations. In contrast, the *T*_onset_ of the EVOs/DTBA reactions occur at higher temperatures, compared to those of DPA formulations (Table 1). 

Thereafter, the comparison between aliphatic and aromatic hardeners shows that the first one is less reactive than DTBA and DPA, in terms of reaction enthalpy.

Considering these results on EVO reactivities with these hardeners, the curing and post-curing protocols were established accordingly for each formulation considering the reactions enthalpy and temperatures range, as summarized in Table S2. As noticed by the DSC study, ESFO formulations require a longer curing protocol than the other EVOs crosslinked with the same hardener. 

The DDA thermosets were prepared by curing in the oven, following the protocols given in Table S2. The completion of the curing process was confirmed by the DSC analysis of the prepared thermosets, which had no residual enthalpy during the second heating program.

### 3.2. Curing Kinetic Analysis

**Figure 2:**
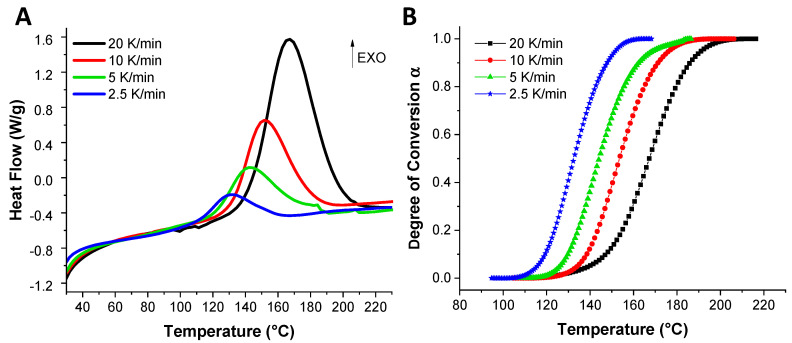
DSC thermograms obtained at indicated heating rates for the reference ELO/DTBA system (**A**) and degrees of conversion dependence with temperature (**B**).

To determine the kinetic parameters of the EVOs reaction with the dicarboxylic acids, dynamic DSC analyses were performed at 2.5, 5, 10 and 20 °C·min^−1^ heating rates. Figure 2A illustrates the obtained DSC thermograms in function of the heating rate for the reference system, ELO/DTBA. The DSC thermograms were converted into degree of conversion vs. temperature in function of the heating rate and the dependence is given in Figure 2B.

Kissinger [26] and Ozawa [27] kinetic methods were used to calculate the activation energy (*E*_a_) and the pre-exponential factor (*A*) of the cure of the epoxy resin examined in this study. Based on Kissinger’s method, the activation energy could be obtained from the peak temperatures at different heating rates using Equation (3):

(3) $$\ln\left(\frac{\beta }{T_{peak}^{2}}\right)=\ln\left(\frac{AR}{E_{a}}\right)+\frac{E_{a}}{RT_{peak}}$$

where *R* is the universal gas constant, *β* is the heating rate and *T_peak_* is the temperature of the exothermic peak. Ozawa’s method can be expressed by Equation (4):

(4) $$\ln\beta =-1.052\;\cdot \left(\frac{E_{a}}{RT}\right)+\ln\left(\frac{AE_{a}}{R}\right)-\ln\left(F\left(x\right)\right)-5.331$$

where *F*(x) is a conversion-dependent term. By plotting ln (β/*T*^2^_*peak*_) vs. 1/*T*_*peak*_ (Kissinger) and ln(*β*) vs. 1/*T*_*peak*_ (Ozawa), the activation energy and the pre-exponential factor can be obtained by calculating the slope of the linear fit and the intercept, respectively. 

**Figure 3:**
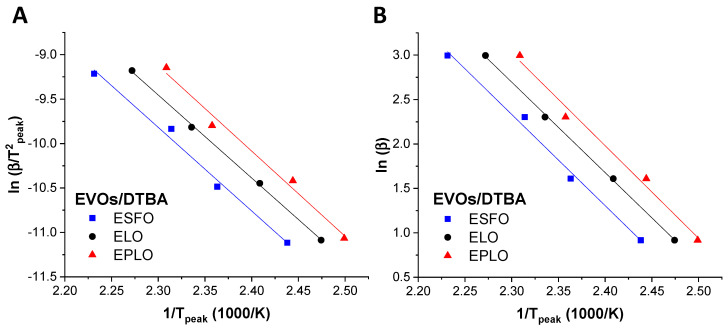
Linear plot based on (**A**) the Kissinger equation and (**B**) the Ozawa kinetic method for the EVOs/DTBA curing systems.

The use of both methods leads to a good linearity (Figure 3) for all the EVOs/DTBA studied curing systems. Using both methods, the lowest values of *E*_a_ were obtained for EPLO, 76.72 and 83.65 kJ·mol^−1^, confirming again that this EVO has the highest reactivity (Table 2). Recently, we calculated the *E*_a_ applying in situ FT-IR analysis during the curing of EVOs with DTBA and DPA and also reported by this method that EPLO is the most reactive monomer, with a lower *E*_a_ value, as a consequence of its high epoxy content [28]. The ESFO/DTBA system shows the highest *E*_a_ values, 79.23 and 86.37 kJ·mol^−^^1^ (Table 2), in good agreement with the previous DSC results related to the enthalpy of this reaction. 

The kinetic evaluation was further applied to evaluate the effect of the diacid hardeners on their curing reactivity with ELO. In this series, the DTBA showed again the lowest activation energy, ~77.65 kJ·mol^−1^. This result corroborates well with our previous kinetic investigation for ELO with DTBA and DPA [28]. A higher *E*_a_ was obtained for the formulation with the DPA hardener, so not containing the disulphide linkage, ~81.65 kJ·mol^−1^. We explained that the presence of dynamic S–S bonds in the *β* position of the carboxylic group increases the reactivity of the acidic proton toward the epoxy groups, and therefore produce an increase in reactivity [28]. In addition, the comparison between aromatic vs. aliphatic hardener (ELO/DTBA vs. ELO/DTDA) exhibited the lowest reactivity for the aliphatic hardener, with, respectively, 77.65 vs. 108.53 kJ·mol^−1^. 

Zhou et al. [29] correlated the low value of *E*_a_ to a faster thermal recyclability and repairability rate for the lignin-derived polycarboxylic acid (L-COOH) with a poly (ethylene glycol) diglycidyl ether (PEG-epoxy). 

Regarding the DDA reactivity with ELO, we obtained very similar results with those reported by Ding et al. [30]. The authors reported for ELO/DDA curing in the presence of DMAP as an initiator, *E*_a_ ~79.2 kJ·mol^−1^, while our value is ~80.44 kJ·mol^−1^.

The pre-exponential factor (*A*), also called the frequency factor is the pre-exponential constant. The calculated values are given in Table 2.

**Figure 4:**
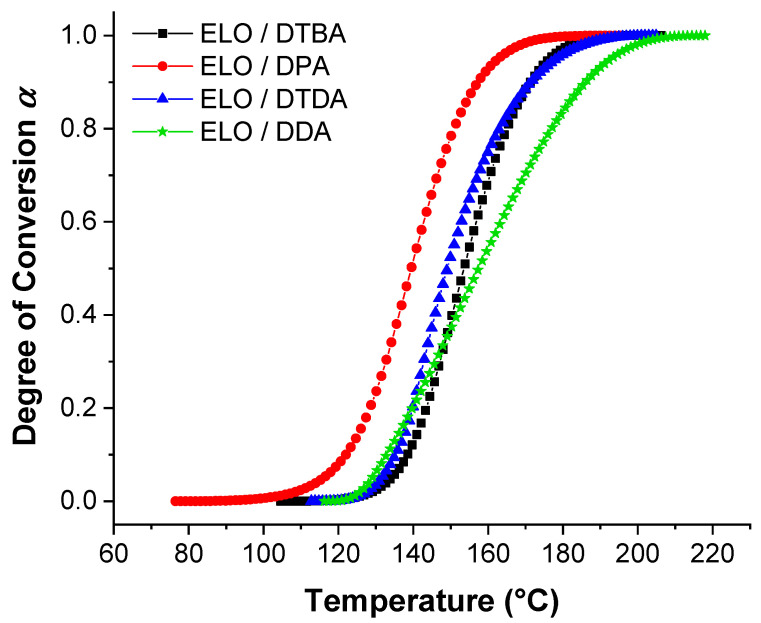
Degree of conversion vs. temperature for the reference resin in function of the crosslinkers.

Figure 4 displays the evolution of the degree of conversion in function of the temperature for ELO curing formulations, varying the crosslinkers. From this figure, we can notice that ELO/DPA is the system that starts the reaction at the lowest temperature (100–110 °C) and reaches the full conversion also at the lowest temperature (~160 °C) than the other systems. This result is in good agreement with those already discussed and shown in Table 1 and Figure 1. The DTDA crosslinker that gives the highest *E*_a_ with ELO (~108.53 kJ·mol^−1^, Table 2), exhibits the comparable evolution of the degree of conversion vs. temperature as DTBA. This result can be correlated and explained by the electron-donor character of the aliphatic chain of DTDA that increases the electron density in the carboxylate anion (RCOO^−^) and therefore decreasing the formation of reactive species. In contrast, DTBA, as demonstrated by our team, increases the selectivity control for the propagation reaction between epoxy acid with the carboxylic–carboxylate complex formation [31].

### 3.3. Thermal and Mechanical Properties of the EVOs-Based Epoxy Resins in Function of the Crosslinker Structures

The thermo-mechanical properties of the obtained thermosets were evaluated in order to discuss the effect of the crosslinkers with/without the S–S bonds. Moreover, the recycling of these thermosets was applied, and the recycled materials tested. The protocols used for recycling are summarized in Table S2, while the morphological aspect of reprocessed materials are given in Table S3. We should highlight that the shorter time for reprocessing was needed for the thermosets obtained with hardeners containing S–S bonds. This result can be correlated to the dual recycling process that acts in the disulphide hardeners: S–S metathesis and transesterification [7,10].

### 3.4. T_g_ Investigation by DSC Analysis of Virgin and Recycled Thermosets

**Figure 5:**
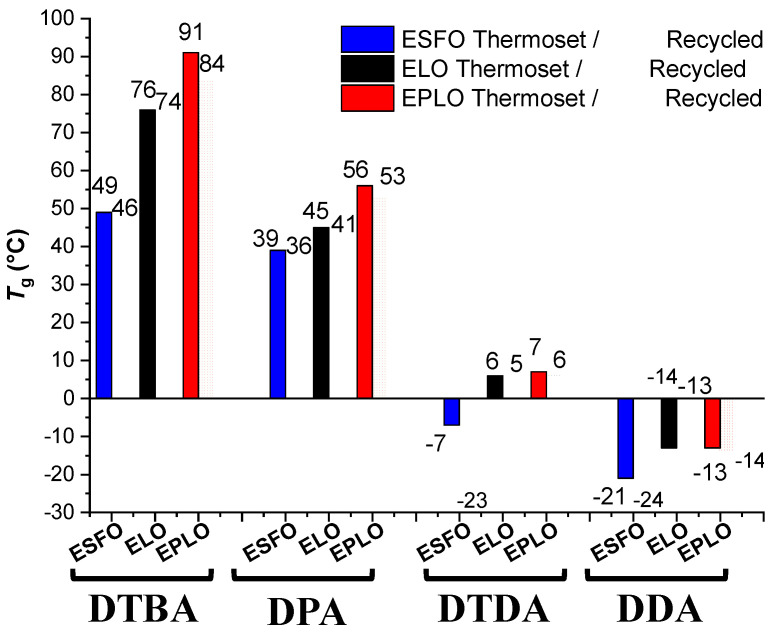
Glass transition of the virgin and recycled EVOs-based thermosets in function of the formulation.

The prepared EVOs thermosets were submitted to mechanical recycling (reprocessing) and the thus obtained recycled materials were characterized by DSC analysis to determine the *T_g_* temperature values. In this manner, both the virgin and recycled thermosets were compared [22]. The thermosets prepared with DTBA show the temperature of this transition from ~49 °C, in the ESFO/DTBA resin, to 91 °C, for EPLO/DTBA. In contrast, the aromatic hardener without S–S linkages, DPA, gave materials with much lower *T_g_*s, respectively, 39 °C (ESFO/DPA) or 56 °C (EPLO/DPA) (Figure 5). It seems that the networks with disulphide bridges have restricted chain motions compared to the other networks. Similar results were reported by Ma et al. [10], in isosorbide-derived epoxy (IS-EPO) crosslinked with an aromatic disulphide diamine (4-AFD) or with a curing agent without S–S bonds (MDA; 4,4′-methylenedianiline). The authors reported higher glass transition values when the S–S bonds were included in the networks, i.e., 40.6 °C for IS-EPO/4-AFD and 37.3 °C IS-EPO/MDA with a decreasing *T_g_* after the recycling process.

The incorporation of flexible segments in the structure of reaction’s components conduct to softer materials, with lower *T_g_*. DTDA- and DDA-based thermosets exhibit lower *T_g_* values, confirming that the aliphatic chains increase the mobility of the chains’ networks. 

In the series of thermosets produced with hardeners containing S–S bonds, the glass transition temperatures decrease from 91 °C (EPLO/DTBA) to −7 °C (ESFO/DTDA). 

A strong decrease in *T_g_* was obtained for the networks based on aliphatic hardener without disulphide bridges, DDA. The lowest *T_g_* value of about –21 °C was obtained for the ESFO/DPA material. DDA with higher aliphatic length than DTDA produces thermosets with the lowest *T_g_*.

The recycled thermosets exhibit decreased values of the *T_g_*, compared to the virgin ones, the most affected being the ESFO/DTDA thermoset, decreasing from −7 to −23 °C.

### 3.5. Dynamic Mechanical Properties

**Figure 6:**
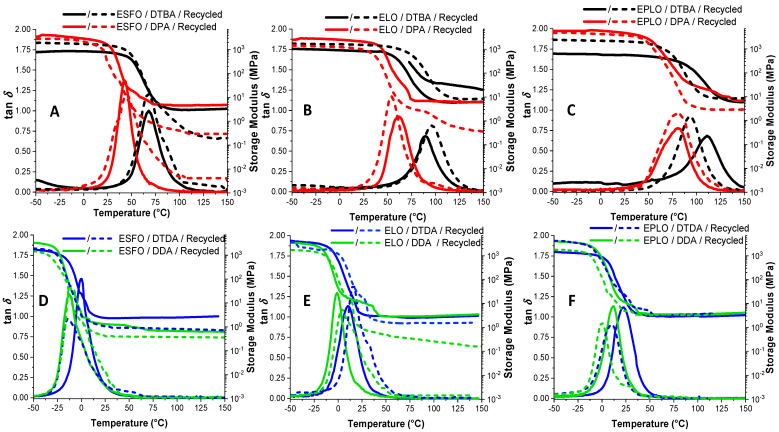
Evolution of tan δ with temperature for the virgin and recycled EVOs thermosets obtained with aromatic (**A**–**C**) or aliphatic (**D**–**F**) crosslinkers.

Figure 6 presents the temperature dependence of loss factor (tan *δ*) and of the storage modulus (*E*’) of the virgin and recycled systems formulated with the different crosslinkers. The DMA data are tabulated in Table 3. The α-transition temperatures were determined as the maximum of the tan *δ* curves. The obtained values display the same trend as in DSC, showing that the S–S dynamic bonds produce networks with higher *T_g_*. This analysis shows that the tan *δ* values are ranging from 68 to 111 °C in the networks based on DTBA and from 43 to 82 °C for DPA, in function of the EVOs epoxy content (Figure 6A–C). The values obtained for EPLO/DTBA virgin thermosets, ~111 °C, are comparable with those for DGEBA/4-AFD vitrimers reported by Odriozola et al. [4], ~130 °C. 

As seen in Figure 6, the shape and the amplitude of the tan *δ* curves is modified for the recycled thermosets, predicting in some case a decrease in crosslink densities. Therefore, we can observe that the amplitude of the tan *δ* is higher in the case of recycled materials (excepting EPLO/aliphatic-based hardeners) and at the same time the Δ*T* range in which the transition occurs is also larger.

In Figure 6D–F, we can observe that the thermosets prepared with aliphatic hardeners (DDA or DTDA) display the α-transitions at lower temperature, from 22 °C for EPLO/DTDA to 0.5 °C for ESFO/DTDA. Chen et al. [8] reported similar values of ~30 °C for vitrimers based on DGEBA/DTDA cured in presence of triazobicyclodecene (TBD). DDA was also used in the literature as a curing agent for the epoxidized broccoli oil (EBO) by Gobin et al. [32]; the prepared thermosets having tan *δ* = −2.3 °C. 

Curves of the storage modulus vs. temperature presented higher moduli in the glassy state for the resins without the dynamic crosslinkers (DPA and DDA) according to the literature, with comparable modulus values in the rubbery plateau [10]. The calculation made by Equation (1) allowed the calculation of the crosslinking density. The EPLO/DTBA thermoset was confirmed as the resin with the highest crosslinking, with 3.0 mmol·cm^−3^. In almost all cases, higher crosslink density was calculated for the EVOs cured with dynamic crosslinkers, with higher values in the presence of DTBA or DPA.

As seen in Table 3, one of the most affected parameters after the reprocessing was the crosslinking density. In the case of the ESFO/DPA system, the recycling produces a reduction from 0.5 to 0.11 mmol·cm^−3^ and for EPLO/DTBA from 3.0 to 1.0 mmol·cm^−3^. ELO and EPLO/DPA recycled thermosets displayed a smaller decrease, from 0.68 to 0.34 mmol·cm^−3^ for the reference and from 1.00 to 0.68 mmol·cm^−3^ for EPLO. These results can be explained by the chemical rearrangements occurred during the reprocessing. The systems with hardeners containing S–S dynamic bonds suffer dual dynamic reorganization by S–S exchange and by transesterifications that retain network crosslinking after the recycling process. EPLO-based thermosets with initial high crosslink density are losing a part of the network connectivity, perhaps due to the competition between network reorganization kinetics and those of the S–S metathesis and transesterification, respectively.

For the thermosets based on aliphatic crosslinkers, the decreasing network density after the recycling shows a small diminution. For example, ESFO/aliphatic crosslinker thermosets present the most significant decrease in crosslink density, from 0.29 to 0.18 mmol·cm^−3^ with DTDA and from 0.16 to 0.11 mmol·cm^−3^ with DDA. The decrease in the *T_g_*, modulus and consequently the crosslinking density of the networks after recycling is in contrast with the materials’ density that in the majority of cases increases after reprocessing (Table 3). These trends can be related with the molecular motions, and the formation of a more compact network during recycling. 

### 3.6. Thermal Stability 

**Figure 7:**
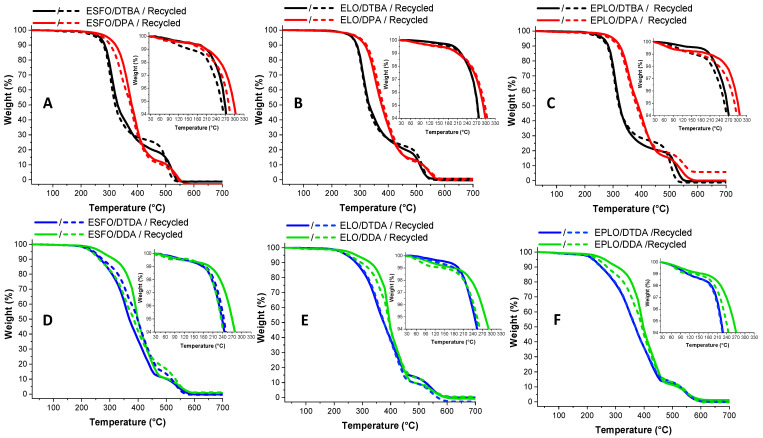
TGA analyses and comparison between the virgin and recycled resins, aromatic (**A**–**C**) or aliphatic (**D**–**F**) crosslinkers. Heating at 10 °C/min, under air.

The thermal stability of the virgin/recycled thermosetting resins were investigated by TGA under air (Figure 7A–F). The DTG curves (Figure S4) clearly show that the resins display two stages of thermal decomposition. All the virgin and reprocessed thermosets show a good thermal stability, with the onset of pyrolysis at ~200 °C. The highest *T*_5%_ can be observed in the case of thermosets based on aromatic hardener without disulphide linkage, DPA, with values ranging between 295 and 300 °C (Table 4). In contrast, the dynamic and labile character of S–S bonds produces a diminution of thermal stability. The explanation of the lower thermal stability of S–S bonds is related to its dissociation energy [32,33]: ~429 kJ·mol^−1^ compared with that of C–C linkages with an energy of around 607 kJ·mol^−1^. Therefore, the thermosets based on DTBA show a decomposition temperature in the range of 255–275 °C, without a relevant decrease in properties after the mechanical reprocessing. These values of thermal degradation are in line with the reported results for isosorbide-derived epoxy cured with the diamine crosslinkers containing or not disulphide bonds [10]. Guo et al. [34] also reported comparable data for the bis-disulphide dynamic epoxy resins obtained from the synthetized bis (4-glycidyloxyphenyl) disulphide crosslinked with 4-AFD. 

In DDA-based thermosets, the fact that the hardener contains two more C–C bonds provokes an increased stability compared to DTDA, by the fact that DDA chains can absorb and dissipate more energy. Ding et al. [30] reported this evolution in ELO thermosets prepared with dicarboxylic acids (DCAs) having different lengths, showing higher thermal stability in function of the length of the crosslinker.

No decreasing in *T*_5%_ was observed in the recycled resins prepared with disulphide containing hardeners, DTBA and DTDA. The values reported in Table 4 highlight that the *T*_5%_ of the dual dynamic thermosets showed constant decomposition temperature for the virgin and reprocessed ones.

The statistic heat-resistant index temperature (*T*_S_) [35,36], is another characteristic parameter to characterize the thermal stability of a material, calculated according to Equation (5):

(5) $$Ts=0.49\;\left[T_{5\%}+0.6\;\left(T_{30\%}-T_{5\%}\right)\right]$$

where *T*_30%_ is the temperatures at 30% weight loss. 

The obtained *T*_S_ values summarized in Table 4 are in good agreement with those of DGEBA/MHHPA and DGEBA/D230 (poly(propylene glycol) bis(2-aminopropyl ether)) thermosets [37] with *T*_S_ around 180 °C or with those of epoxidized sucrose soyate (ESS)/citric or malic acids [36], *T*_S_~150 °C.

### 3.7. Solvent Stability

**Figure 8:**
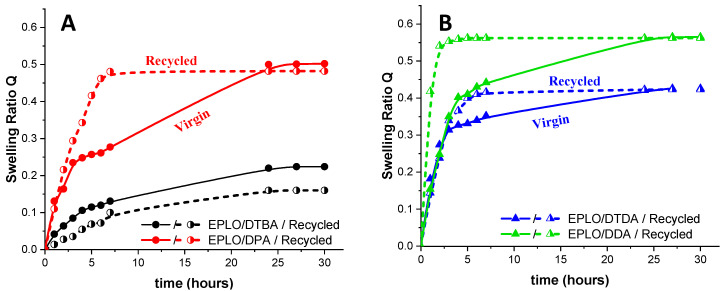
Swelling experiments in trichlorobenzene for the virgin and recycled EPLO thermosets prepared with (**A**) aromatic (DTBA and DPA) and (**B**) aliphatic (DTDA and DDA) crosslinkers.

The swelling abilities of the virgin/recycled resins were exemplified for EPLO thermosets. The experiments were performed in trichlorobenzene at room temperature and the swelling ratio was calculated according the Equation (2). The obtained results are illustrated in Figure 8. The resins made with S–S dynamic crosslinkers swell less than the ones without disulphide linkages, reaching equilibrium after 10 h. As confirmed by the calculation of the crosslinking density, DDA-based thermosets display higher swelling, while the DTBA ones are less affected at the end of the experiment. No degradation or dissolution of the samples was detected during this experiment.

**Figure 9:**
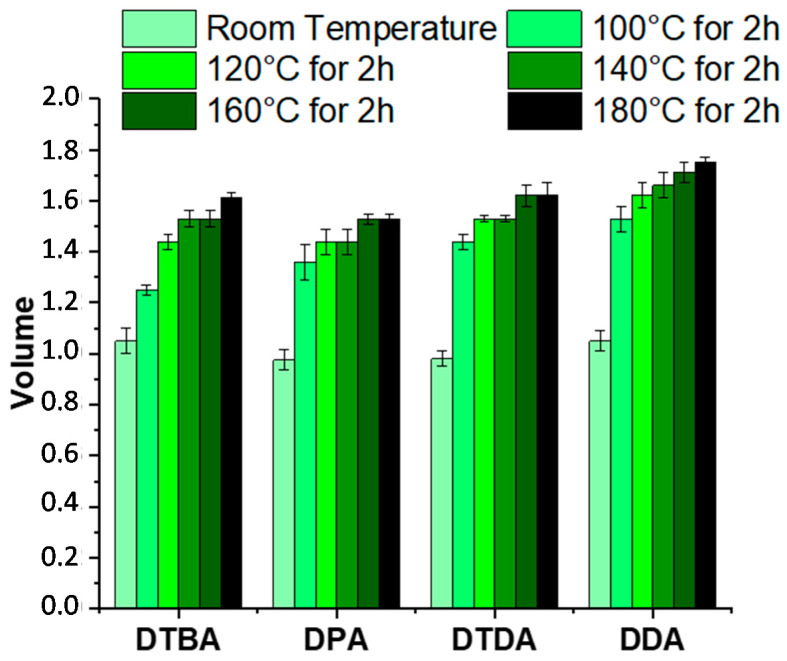
Evolution of the swelling volume of EPLO resins at room temperature and after the immersion steps in trichlorobenzene from 100 to 180 °C.

The solvent stability was also tested in the same solvent, in function of the temperature. All the thermosets were insoluble even when immersed in trichlorobenzene and heated from 100 to 180 °C (Figure 9 and Figure S5). At a first step, the samples were kept at room temperature, for 2 h, then the temperature was increased to 100 °C, kept again 2 h, thereafter heated with isotherm steps of each 20 °C for 2 h until 180 °C. The aspects of the samples are displayed in Figure S5. 

The swelling of the samples was determined for each sample by measuring their dimensions after each immersing step. The thermoset volume was measured, assuming the thickness constant (Figure 9). DDA- and DTDA-based thermosets exhibit an increase of ~60% in volume at the end of the test, with a slight gain with the aromatic diacid, 63% with DPA and 65% with DTBA, respectively. However, the resins are insoluble and stable in trichlorobenzene in the large range of temperatures.

## 4. Conclusions

Reprocessable networks were obtained based on three EVOs crosslinked with aromatic and aliphatic carboxylic diacids. The hardener structure plays a fundamental role in the design of the thermoset and moreover on its properties. This work shows that disulphide linkages increase the reactivity of the aromatic or aliphatic diacids vs. EVOs. Moreover, the EVOs thermosets derived from these hardeners have superior values of glass transition, compared to their homologous counterparts that do not contain S–S bonds. In contrast, these resins showed lower thermal stability, due to the presence of labile disulphide bonds. All the bio-thermosets exhibit excellent recyclability properties combined with solvent resistance even at high temperatures. The concept of synthesis of these bio-based recycled materials can be used to obtain many alternatives of low-cost resins with reprocessing abilities.

## Figures and Tables

**Table 1:** DSC results for the EVOs crosslinked with DTBA, DPA and DTDA at R_e/a_ = 1.

EVOs	Crosslinker	*T*_peak_ (°C)	Reaction Interval (°C)	Δ*H* (J/g)
**ESFO**	DTBA	159	125–195	172 ± 2
	DPA	144	110–180	147 ± 2
	DTDA	148	132–220	125 ± 2
**ELO**	DTBA	155	118–197	197 ± 2
	DPA	138	105–181	157 ± 2
	DTDA	143	120–218	162 ± 2
**EPLO**	DTBA	151	118–197	207 ± 2
	DPA	134	100–180	173 ± 2
	DTDA	141	115–205	172 ± 2

**Table 2:** Kinetic parameters determined by fitting non-isothermal DSC data.

Cured Resin	*E*_a_ (kJ/mol) Kissinger	*E*_a_ (kJ/mol) Ozawa	*A* (s^−1^) Kissinger
**ESFO/DTBA**	79.23	86.37	1.29 × 10^6^
**ELO/DTBA**	77.65	84.66	1.56 × 10^6^
**EPLO/DTBA**	76.72	83.65	3.95 × 10^6^
**ELO/DPA**	81.65	88.37	1.70 × 10^7^
**ELO/DTDA**	108.53	115.34	3.14 × 10^10^
**ELO/DDA**	80.44	87.65	1.61 × 10^6^

**Table 3:** Properties of the virgin and recycled thermosets.

EVOs	Hardener	tan *δ*^(A)^ (°C)		*E*’ Glassy State ^(A)^ (MPa)		*E*’ Rubbery State ^(A)^ (MPa)		Crosslinking Density ^(A)^ (mmol/cm^3^)		Density ^(B)^ (g/cm^3^)	
		V *	R **	V *	R **	V *	R **	V *	R **	V *	R **
**ESFO**	**DTBA**	68 ± 1	79 ± 1	780	1920	3.13	0.32	0.31	0.27	1.12	1.10
	**DPA**	43 ± 1	45 ± 1	3400	2810	4.88	0.19	0.50	0.11	0.91	0.98
	**DTDA**	0.5 ± 1	−14 ± 1	1710	1865	2.74	0.22	0.29	0.18	1.15	1.19
	**DDA**	−12 ± 1	−8 ± 1	3300	1470	0.61	0.13	0.16	0.11	1.06	1.13
**ELO**	**DTBA**	91 ± 1	97 ± 1	1560	2550	8.6	8.9	1.25	0.82	1.02	1.10
	**DPA**	62 ± 1	79 ± 1	2500	4000	6.54	5.81	0.68	0.58	0.94	0.99
	**DTDA**	10 ± 1	15 ± 1	5000	3640	2.55	1.60	0.34	0.33	1.17	1.15
	**DDA**	−1 ± 1	−5 ± 1	3800	1470	3.53	0.32	0.36	0.15	1.13	1.15
**EPLO**	**DTBA**	111 ± 1	92 ± 1	630	2400	5.98	9.15	3.00	0.82	0.99	1.02
	**DPA**	82 ± 1	83 ± 1	4660	5620	2.95	7.44	1.00	0.68	0.95	0.97
	**DTDA**	22 ± 1	10 ± 1	1680	4250	3.30	3.81	0.35	0.38	1.00	1.14
	**DDA**	12 ± 1	0 ± 1	5030	1780	4.14	3.69	0.43	0.40	0.96	1.19

**^(A)^** determined by DMA, **^(B)^** determined by the mass/volume ratio, * virgin, ** recycled.

**Table 4:** Thermal stability data, T_5%_ and statistic heat-resistant index temperature (*T*_S_) of virgin and recycled thermosets.

EVOs	Hardeners	*T*_5%_ Virgin (°C)	*T*_5%_ Recycled (°C)	*T*_S_ Virgin (°C)	*T*_S_ Recycled (°C)
**ESFO**	**DTBA**	265	265	164	160
	**DPA**	295	280	175	170
	**DTDA**	235	235	167	175
	**DDA**	270	230	180	172
**ELO**	**DTBA**	275	275	167	165
	**DPA**	295	295	178	178
	**DTDA**	245	245	171	170
	**DDA**	275	255	179	175
**EPLO**	**DTBA**	255	250	160	161
	**DPA**	300	290	176	171
	**DTDA**	220	220	164	165
	**DDA**	260	240	177	173

## Supplementary Materials

The following are available online at https://www.mdpi.com/2073-4360/13/4/534/s1, Figure S1. DSC thermograms during EVOs curing with DTBA at different ratios. Heating at 10 °C·min^−1^; Figure S2. DSC thermograms of EVOs/DPA, curing reaction at different ratios, during heating at 10 °C·min^−1^; Figure S3. DSC thermograms of the EVOs/DTDA curing reaction, at different ratios, during heating at 10 °C·min^−1^; Figure S4. DTG of the virgin and recycled resins with EVOs combined with the four crosslinkers; Figure S5. Aspect of the EPLO resins at room temperature and after the swelling experiment from 100 to 180 °C; Table S1. Structures and characteristics of the selected reagents; Table S2. Curing and reprocessing conditions for the EVOs combined with the different crosslinkers; Table S3. Aspect of the recycled resins in function of formulation; Table S4. DSC results for the EVOs/DTBA curing reaction, in function of the ratio R_e/a_; Table S5. DSC results for the EVOs/DPA curing reaction, in function of the ratio R_e/a_; Table S6. DSC results for the EVOs/DTDA curing reaction, in function of the ratio R_e/a_.

## Abbreviations

EVO—epoxidized vegetable oil; ELO—epoxidized linseed oil; ESFO—epoxidized safflower oil; EPLO—epoxidized perilla oil; DTBA—2,2’-dithiodibenzoic acid; DPA—diphenic acid; DTDA—3,3′-dithiodipropionic acid; DDA—dodecanedioic acid; IM—imidazole; DSC—differential scanning calorimetry; DMA—dynamic mechanical analysis; TGA—thermogravimetric analysis; DTG—derivative thermogravimetric analysis; *T*_g_—glass transition temperature; vs.—versus; DCAs—dicarboxylic acids, *E*_a_—activation energy; *A*—pre-exponential factor.
