# Supplementary material for: Influence of the Presence of Disulphide Bonds in Aromatic or Aliphatic Dicarboxylic Acid Hardeners Used to Produce Reprocessable Epoxidized Thermosets
Source: Polymers (Basel). 2021 Feb 11;13(4):534. doi: 10.3390/polym13040534 (PMC7918345; doi:10.3390/polym13040534)
Supplement: Supplementary file 1 [file polymers-13-00534-s001.pdf]

polymers

MDPI

Supplementary Materials

Influence of the Presence of Disulphide Bonds in Aromatic or Aliphatic Dicarboxylic Acid Hardeners Used to Produce Reprocessable Epoxidized Thermosets

Chiara Di Mauro and Alice Mija *

Université Côte d'Azur, Institut de Chimie de Nice, UMR CNRS 7272, 28 Avenue Valrose, 06108 Nice, France; Chiara.DI-MAURO@univ-cotedazur.fr
* Correspondence: Alice.MIJA@univ-cotedazur.fr

Citation: Di Mauro, C.; Mija, A. Influence of the Presence of Disulphide Bonds in Aromatic or Aliphatic Dicarboxylic Acid Hardeners Used to Produce Reprocessable Epoxidized Thermosets. *Polymers* **2021**, *13*, 534. https://doi.org/10.3390/polym13040534

Academic Editor: Viera Khunová
Received: 25 January 2021
Accepted: 9 February 2021
Published: 11 February 2021

Publisher's Note: MDPI stays neutral with regard to jurisdictional claims in published maps and institutional affiliations.

Copyright: © 2021 by the authors. Submitted for possible open access publication under the terms and conditions of the Creative Commons Attribution (CC BY) license (http://creativecommons.org/licenses/by/4.0/).

Index of Figure and Table

Figure S1. DSC thermograms during EVOs curing with DTBA at different ratios. Heating at 10 °C.min–1. 4

Figure S2. DSC thermograms of EVOs/DPA curing reaction, at different ratios, during heating at 10 °C.min^{-1}. 4.

Figure S3. DSC thermograms of EVOs/DTDA curing reaction, at different ratios, during heating at 10 °C.min^{-1}. 5.

Figure S4. DTG of the virgin and recycled resins with EVOs combined with the four crosslinkers. 6.

Figure 5. Aspect of the EPLO resins at room temperature and after the swelling experiment from 100 to 180 °C 6.

Table S1. Structures and characteristics of the selected reagents. 1.

Table S2. Curing and reprocessing conditions for the EVOs combined with the different crosslinkers. 3.

Table S3. Aspect of recycled resins in function of formulation 3.

Table S4. DSC results for EVOs/ DTBA curing reaction, in function of the ratio $R_{e/a}$ 4.

Table S5. DSC results for EVOs/ DPA curing reaction, in function of the ratio $R_{e/a}$ 5.

Table S6. DSC results for EVOs/ DTDA curing reaction, in function of the ratio $R_{e/a}$ 5.

Table S1. Structures and characteristics of the selected reagents.

EVOs	Structure	Epoxy Content (meq/g)	Mw (g/mol)
**ESFO**	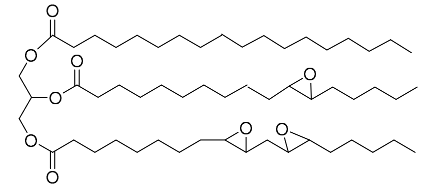 **Epoxidized Safflower Oil**	5.06	960.2
**ELO**	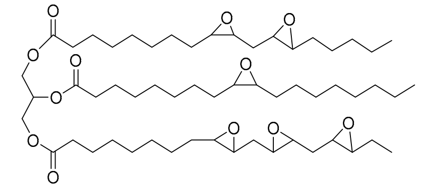 **Epoxidized Linseed Oil**	5.61	980
**EPLO**	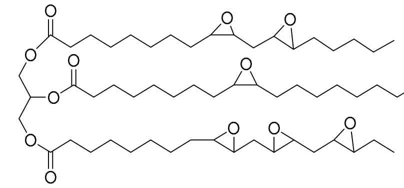 **Epoxidized Perilla Oil**	6.50	986.4
		**Melting temperature (°C)**	**Mw (g/mol)**
**DTBA**	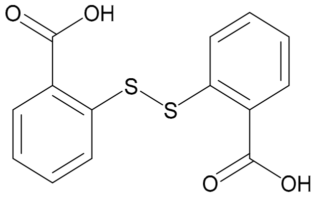 **2,2′-Dithiodibenzoic acid**	287–290	306.35
**DPA**	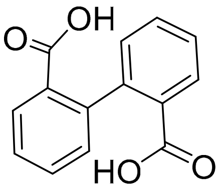 **Diphenic acid**	227–229	242.23
**DTDA**	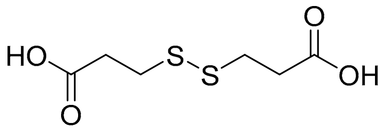 **3,3′-Dithiodipropionic acid**	155–158	210.27
**DDA**	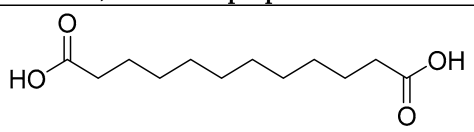 **Dodecanedioic acid**	127–129	230.30
**IM**	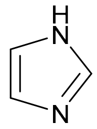 **Imidazole**	89–91	68.07

Table S2. Curing and reprocessing conditions for the EVOs combined with the different crosslinkers.

EVOs	*Hardener*	*Curing*	*Post-Curing*	*Recycling Conditions*
**ESFO**	**DTBA**	140 °C–60 min	170 °C–30 min	170 °C–10 min-2 tons
	**DPA**	140 °C–60 min	180 °C–60 min	170 °C–90 mins-2 tons
	**DTDA**	140 °C–60 min	180 °C–60 min	170 °C–10 min-2 tons
	**DDA**	140 °C–60 min	180 °C–60 min	170 °C–40 min-2 tons
**ELO**	**DTBA**	130 °C–60 min	170 °C–30 min	170 °C–10 min-2 tons
	**DPA**	130 °C–60 min	170 °C–30 min	170 °C–150 min-2 tons
	**DTDA**	140 °C–60 min	180 °C–30 min	170 °C–10 min-2 tons
	**DDA**	140 °C–60 min	18 0°C–30 min	170 °C–40 min-2 tons
**EPLO**	**DTBA**	130 °C–60 min	180 °C–30 min	170 °C–10 min-2 tons
	**DPA**	140 °C–60 min	170 °C–30 mins	170 °C–150 min-2 tons
	**DTDA**	140 °C–60 min	170 °C–30 min	170 °C–10 min-2 tons
	**DDA**	140 °C–60 min	170 °C–30 min	170 °C–40 min-2 tons

Table S3.–Aspect of recycled resins in function of formulation.

EVOs	*Crosslinkers*			
	**DTBA**	**DPA**	**DTDA**	**DDA**
**ESFO**	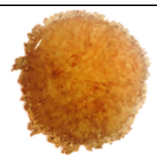	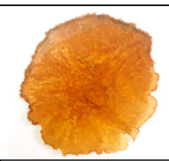	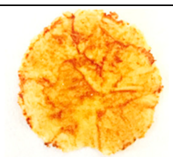	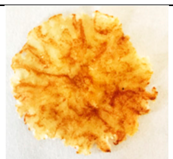
**ELO**	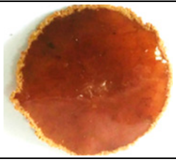	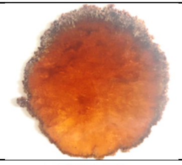	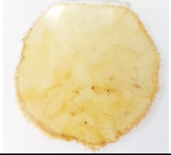	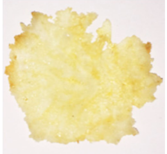
**EPLO**	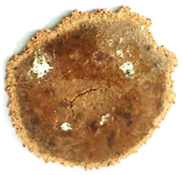	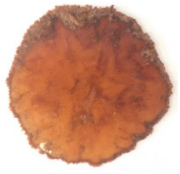	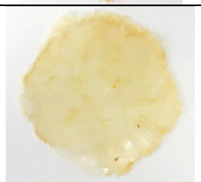	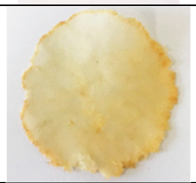

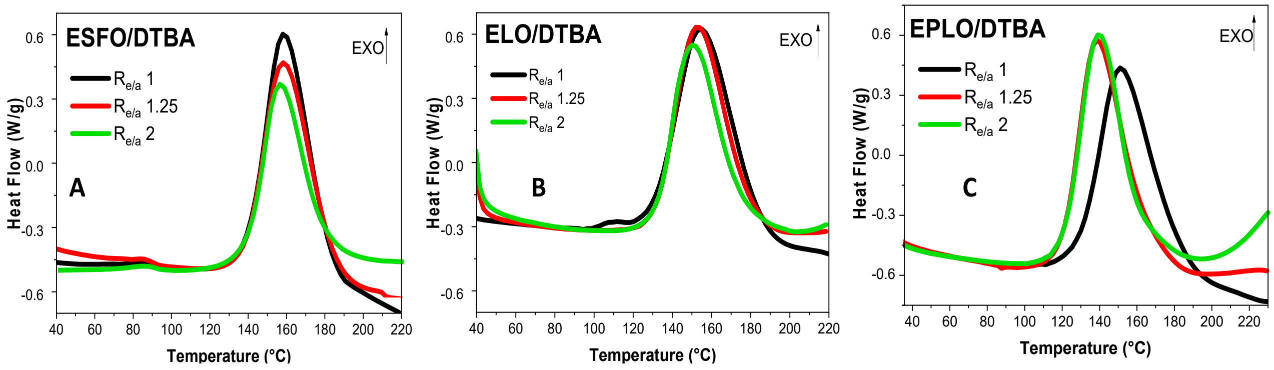

Figure S1.DSC thermograms during EVOs curing with DTBA at different ratios. Heating at 10 °C.min^{-1}.

Table S4. DSC results for EVOs/ DTBA curing reaction, in function of the ratio $\mathbf{R_{e/a}}$.

	Ratio ($R_{e/a}$)	T_{peak} (°C)	Reaction Interval (°C)	Δ*H* (J/g)
**ESFO/DTBA**	1	159	125–195	158
	1.25	158	125–200	169
	2	156	125–190	128
**ELO/DTBA**	1	155	118–197	197
	1.25	154	118–195	190
	2	151	117–193	166
**EPLO/DTBA**	1	151	118–197	217
	1.25	150	104–190	200
	2	150	100–191	191

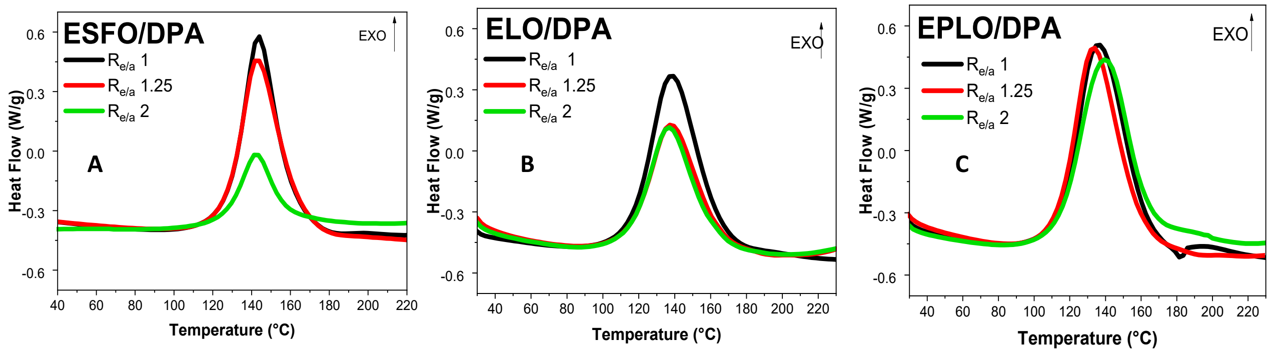

Figure S2. DSC thermograms of EVOs/ DPA curing reaction, at different ratios, during heating at 10 °C.min^{-1}.

Table S5. DSC results for EVOs/ DPA curing reaction, in function of the ratio $\mathbf{R_{e/a}}$.

	Ratio	T_{peak} (°C)	Reaction Interval (°C)	Δ*H* (J/g)
**ESFO/DPA**	1	144	110–180	147
	1.25	143	110–178	140
	2	143	112–165	64
**ELO/DPA**	1	138	105–181	157
	1.25	138	104–185	119
	2	137	105–178	104
**EPLO/DPA**	1	134	100–180	173
	1.25	134	101–190	170
	2	140	101–185	160

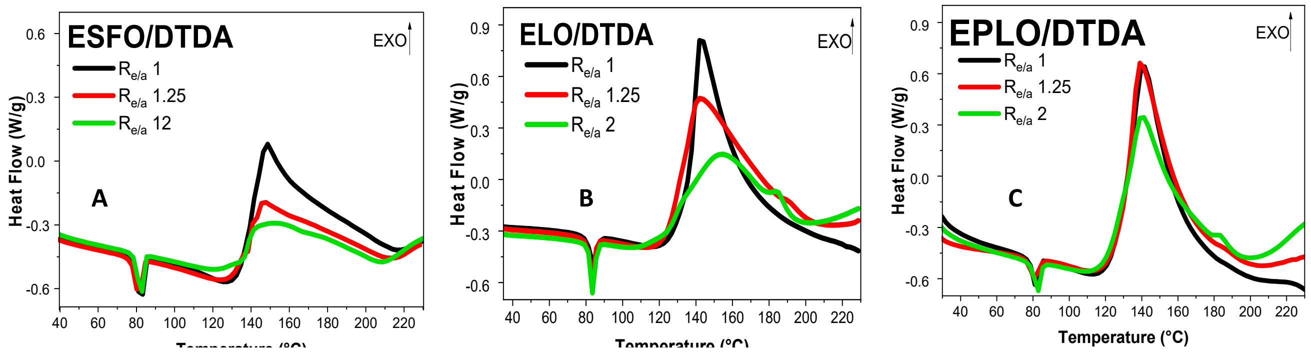

Figure S3. DSC thermograms of EVOs/ DTDA curing reaction, at different ratios, during heating at 10 °C.min^{-1}.

Table S6. DSC results for EVOs/ DTDA curing reaction, in function of the ratio $\mathbf{R}_{e/a}$.

	Ratio	T_{peak} (°C)	Reaction Interval (°C)	Δ*H* (J/g)
**ESFO/DTDA**	1	148	132–220	125
	1.25	147	130–215	121
	2	155	118–208	105
**ELO/DTDA**	1	143	120–218	162
	1.25	142	117–213	155
	2	153	111–195	110
**EPLO/DTDA**	1	141	118–205	172
	1.25	140	115–208	157
	2	140	115–193	135

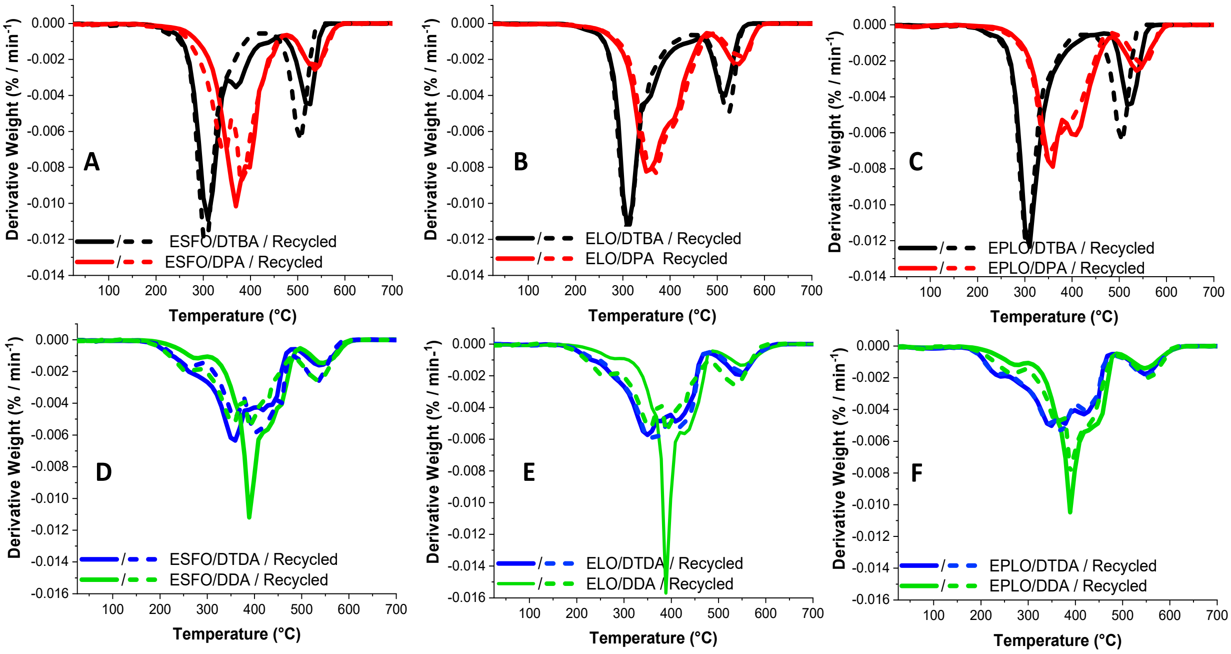

Figure S4. DTG of the virgin and recycled resins with EVOs combined with the four crosslinkers.

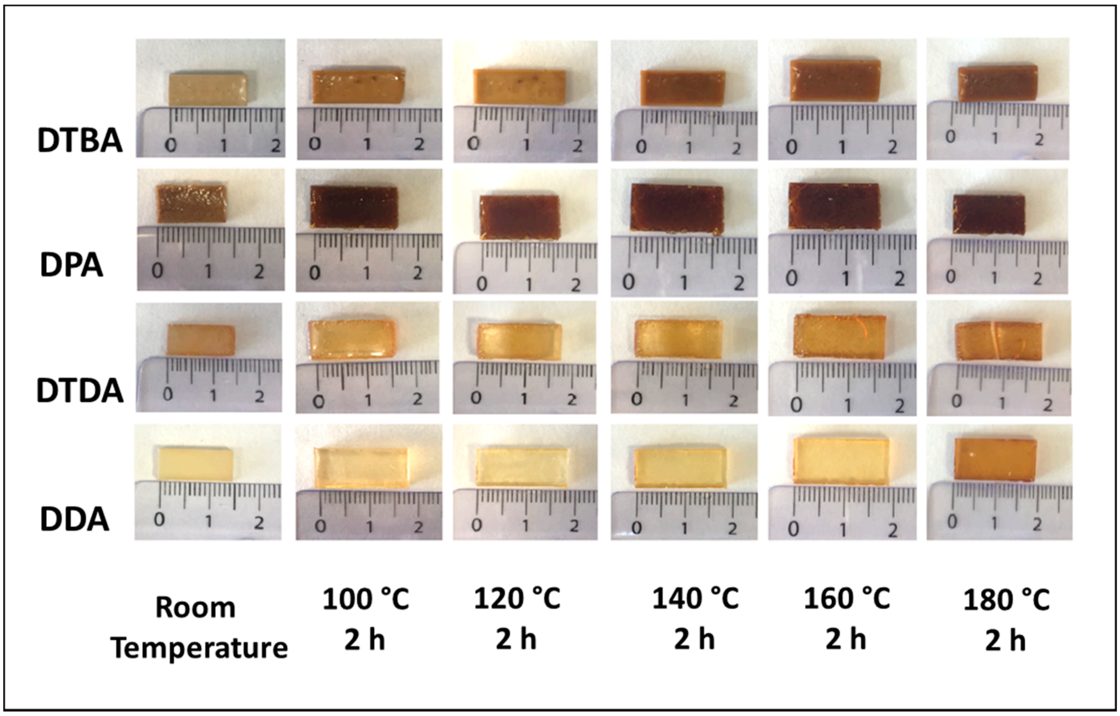

Figure S5. Aspect of the EPLO resins at room temperature and after the swelling experiment from 100 to 180 °C.
